# Stuck in Neutral: Stalled Progress in Statewide Comprehensive Smoke-Free Laws and Cigarette Excise Taxes, United States, 2000–2014

**DOI:** 10.5888/pcd13.150409

**Published:** 2016-06-16

**Authors:** Carissa Baker Holmes, Brian A. King, Stephen D. Babb

**Affiliations:** Author Affiliations: Brian A. King, Stephen D. Babb, Office on Smoking and Health, National Center for Chronic Disease Prevention and Health Promotion, Centers for Disease Control and Prevention, Atlanta, Georgia.

## Abstract

**Introduction:**

Increasing tobacco excise taxes and implementing comprehensive smoke-free laws are two of the most effective population-level strategies to reduce tobacco use, prevent tobacco use initiation, and protect nonsmokers from secondhand smoke. We examined state laws related to smoke-free buildings and to cigarette excise taxes from 2000 through 2014 to see how implementation of these laws from 2000 through 2009 differs from implementation in more recent years (2010–2014).

**Methods:**

We used legislative data from LexisNexis, an online legal research database, to examine changes in statewide smoke-free laws and cigarette excise taxes in effect from January 1, 2000, through December 31, 2014. A comprehensive smoke-free law was defined as a statewide law prohibiting smoking in all indoor areas of private work sites, restaurants, and bars.

**Results:**

From 2000 through 2009, 21 states and the District of Columbia implemented comprehensive smoke-free laws prohibiting smoking in work sites, restaurants, and bars. In 2010, 4 states implemented comprehensive smoke-free laws. The last state to implement a comprehensive smoke-free law was North Dakota in 2012, bringing the total number to 26 states and the District of Columbia. From 2000 through 2009, 46 states and the District of Columbia implemented laws increasing their cigarette excise tax, which increased the national average state excise tax rate by $0.92. However, from 2010 through 2014, only 14 states and the District of Columbia increased their excise tax, which increased the national average state excise tax rate by $0.20.

**Conclusion:**

The recent stall in progress in enacting and implementing statewide comprehensive smoke-free laws and increasing cigarette excise taxes may undermine tobacco prevention and control efforts in the United States, undercutting efforts to reduce tobacco use, exposure to secondhand smoke, health disparities, and tobacco-related illness and death.

## Introduction

Tobacco use causes more than 480,000 premature deaths in the United States annually (1). Smoke-free policies and tobacco taxes reduce tobacco use, increase tobacco use cessation, reduce initiation of tobacco use, and reduce tobacco-related illness and death, including deaths related to exposure to secondhand smoke (1,2). Both population-level interventions contributed, in part, to the major public health achievement of averting 8 million premature smoking-attributable deaths from 1964 through 2014 (3).

The United States has made substantial progress in implementing comprehensive smoke-free laws and cigarette excise taxes (1). Since 2000, the number of states with comprehensive smoke-free laws increased from 0 to 26 states and the District of Columbia (4). Furthermore, cigarette prices increased significantly over the past decade, driven in part by numerous state and local tax increases and a 2009 federal tax increase (1). As of December 31, 2014, the average state cigarette excise tax rate was at an all-time high of $1.54 per pack (5).

Previous studies examined state-specific trends for comprehensive smoke-free laws from 2000 to 2010 and laws that increase cigarette excise tax rates from 2010 to 2011; however, more recent trends have not been assessed (4,6). The objective of this study was to examine state-specific data for both types of laws (smoke-free laws and cigarette excise tax laws) from 2000 through 2014, to determine how implementation of these laws from 2000 through 2009 differs from implementation in more recent years (2010–2014).

## Methods

Data were obtained from the Centers for Disease Control and Prevention’s (CDC’s) State Tobacco Activities Tracking and Evaluation (STATE) system, which contains tobacco-related epidemiologic and economic data and information on state tobacco-related legislation for all 50 US states and the District of Columbia (7). Data are collected quarterly from LexisNexis (RELX Group), an online legal research database, and are then analyzed, coded, and entered into the STATE system (7).

We examined changes in comprehensive state smoke-free laws and state cigarette excise tax laws in effect from January 1, 2000, through December 31, 2014. A law was considered in effect in a specific year if the law was implemented during that year (determined by the date the law took effect, rather than the date the law was enacted). CDC defines a state smoke-free law as comprehensive if it prohibits smoking in private work sites, restaurants, and bars (1,8). To be considered comprehensive, a law must prohibit smoking in all indoor areas in these settings with no exceptions. Some states have laws in effect with less stringent smoking restrictions, such as laws establishing designated smoking areas or permitting smoking in separately ventilated areas. Other states have laws in effect that prohibit smoking at all times in 1 or 2 of these settings, but not all 3; such laws were not considered comprehensive for this assessment, because evidence indicates that they are not as effective as comprehensive smoke-free laws in eliminating population-level exposure to secondhand smoke (1,8). We examined state cigarette excise tax laws in terms of the price per pack; we did not address increases in the tax on other tobacco products (eg, cigars, smokeless tobacco). We used US Census Bureau definitions to categorize states into 4 regions: Northeast, South, Midwest, and West (9).

We assessed changes in state comprehensive smoke-free laws and cigarette excise taxes in effect that occurred from 2000 through 2014, and also compared changes in laws from 2000 through 2009 with changes in laws from 2010 through 2014. We examined the number of laws in effect, changes in state excise tax rates per pack of cigarettes, and the change in the average state excise tax rate per pack over time.

## Results

**Figure 1:**
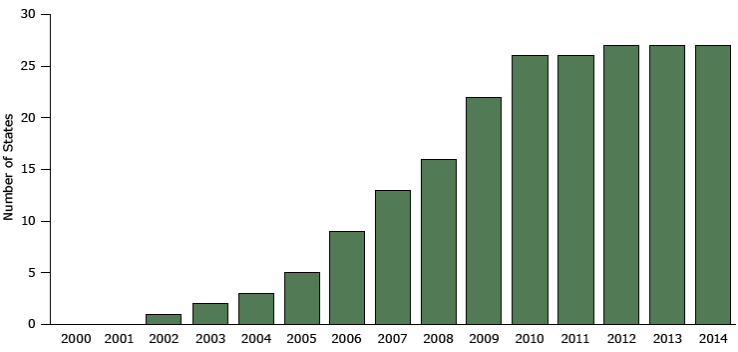
Number of states with comprehensive smoke free laws, 2000–2014. A comprehensive law is one that prohibits smoking at all times in all indoor areas of private work sites, restaurants, and bars. Data are for the year the law went into effect rather than the year it was enacted. Source: Centers for Disease Control and Prevention’s State Tobacco Activities Tracking and Evaluation System. **Table:** 

In 2002, Delaware became the first state to implement a comprehensive smoke-free law. In the following years, several states followed suit, especially during the latter half of the past decade (2005 to 2009). By December 31, 2009, 21 states and the District of Columbia had comprehensive smoke-free laws in effect (Table 1); of these 21 states, 6 had laws go into effect in 2009 (Figure 1).

**Table 1:** Smoke-Free Laws and Cigarette Excise Tax Rates, by State, 2000–2014^a^

State	State Smoke-Free Law		Cigarette Excise Tax				
	Characteristic^a^	Year Law in Effect	Excise Tax Rates	Amount of Tax Increase or Decrease	Year Tax Increase or Decrease Implemented	Percentage Change in Tax, 2000–2009	Relative Percentage Change in Tax, 2010–2014
Alabama	Partial/none	—b	$0.425	$0.260	2004	158	0
Alaska	Partial/none	—b	$1.600	$0.600	2005	100	0
			$1.800	$0.200	2006		
			$2.000	$0.200	2007		
Arizona	Comprehensive	2007	$1.180	$0.600	2002	245	0
			$2.000	$0.820	2006	265	0
Arkansas	Work sites	2006	$0.340	$0.025	2001		
			$0.590	$0.250	2003		
			$1.150	$0.560	2009		
California	Partial/none	—b	$0.870	$0.050	1999	0	0
Colorado	Comprehensive	2006	$0.840	$0.640	2005	320	0
Connecticut	Partial/none	—b	$1.110	$0.610	2002	500	13
			$1.510	$0.400	2003		
			$2.000	$0.490	2007		
			$3.000	$1.000	2009		
			$3.400	$0.40	2011		
Delaware	Comprehensive	2002	$0.550	$0.310	2003	567	0
			$1.150	$0.600	2007		
			$1.600	$0.450	2009		
District of Columbia	Work sites	2006	$1.000	$0.350	2003	285	16
	Comprehensive	2007	$2.000	$1.000	2008		
			$2.500	$0.500	2009		
			$2.900	$0.400	2014		
Florida	Work sites, Restaurants	2003	$1.339	$1.000	2009	295	0
Georgia	Partial/none	—b	$0.370	$0.250	2003	208	0
Hawaii	Comprehensive	2006	$1.200	$0.200	2002	160	23
			$1.300	$0.100	2003		
			$1.400	$0.100	2004		
			$1.600	$0.200	2006		
			$1.800	$0.200	2007		
			$2.000	$0.200	2008		
			$2.600	$0.600	2009		
			$3.000	$0.400	2010		
			$3.200	$0.200	2011		
Idaho	Restaurants	2004	$0.570	$0.290	2003	104	0
Illinois	Comprehensive	2008	$0.980	$0.400	2002	69	102
			$1.980	$1.000	2012		
Indiana	Work sites, restaurants	2012	$0.555	$0.400	2002	542	0
			$0.995	$0.440	2007		
Iowa	Comprehensive	2008	$1.360	$1.000	2007	278	0
Kansas	Comprehensive	2010	$0.700	$0.460	2002	229	0
			$0.790	$0.090	2003		
Kentucky	Partial/none	—b	$0.300	$0.270	2005	1900	0
			$0.600	$0.300	2009		
Louisiana	Work sites, Restaurants	2007	$0.240	$0.040	2000	80	0
			$0.360	$0.120	2002		
Maine	Restaurants, bars	2004	$1.000	$0.260	2001	170	0
	Comprehensive	2009	$2.000	$1.000	2005		
Maryland	Comprehensive	2008	$1.000	$0.340	2002	203	0
			$2.000	$1.000	2008		
Massachusetts	Comprehensive	2004	$1.510	$0.750	2002	230	40
			$2.510	$1.000	2008		
			$3.510	$1.000	2013		
Michigan	Comprehensive	2010	$1.250	$0.500	2002	167	0
			$2.000	$0.750	2004		
Minnesota	Comprehensive	2007	$1.230	$0.750	2005	156	130
			$2.830	$1.600	2013		
Mississippi	Partial/none	—b	$0.680	$0.500	2009	278	0
Missouri	Partial/none	—b	$0.170	$0.040	1993	0	0
Montana	Work sites, restaurants	2005	$0.700	$0.520	2003	844	0
	Comprehensive	2009	$1.700	$1.000	2005		
Nebraska	Comprehensive	2009	$0.640	$0.300	2002	88	0
Nevada	Work sites, restaurants	2006	$0.800	$0.450	2003	129	0
New Hampshire	Restaurants	2007	$0.800	$0.280	2005	242	0
			$1.080	$0.280	2007		
			$1.330	$0.250	2008		
			$1.780	$0.450	2009		
			$1.680	($0.100)	2011		
			$1.780	$0.100	2013		
New Jersey	Comprehensive	2006	$1.500	$0.700	2002	238	0
			$2.050	$0.550	2003		
			$2.400	$0.350	2004		
			$2.575	$0.175	2006		
			$2.700	$0.125	2009		
New Mexico	Comprehensive	2007	$0.910	$0.70	2003	333	82
			$1.660	$0.750	2010		
New York	Comprehensive	2003	$1.110	$0.550	2000	391	58
			$1.500	$0.390	2002		
			$2.750	$1.250	2008		
			$4.350	$1.600	2010		
North Carolina	Restaurants, bars	2010	$0.300	$0.250	2005	800	0
			$0.350	$0.050	2006		
			$0.450	$0.100	2009		
North Dakota	Work sites	2005	$0.440	$0.150	1993	0	0
	Comprehensive	2012					
Ohio	Comprehensive	2006	$0.550	$0.310	2002	421	0
			$1.250	$0.700	2005		
Oklahoma	Partial/none	—b	$1.030	$0.800	2005	348	0
Oregon	Comprehensive	2009	$1.280	$0.600	2002	74	11
			$1.180	($0.100)	2004		
			$1.310	$0.130	2014		
Pennsylvania	Work sites	2008	$1.000	$0.690	2002	416	0
			$1.350	$0.350	2004		
			$1.600	$0.250	2009		
Rhode Island	Comprehensive	2005	$1.000	$0.290	2001	387	1
			$1.320	$0.320	2002		
			$1.710	$0.390	2003		
			$2.460	$0.750	2004		
			$3.460	$1.000	2009		
			$3.500	$0.040	2012		
South Carolina	Partial/none	—b	$0.570	$0.500	2010	0	714
South Dakota	Work sites	2002	$0.530	$0.200	2003	364	0
	Work sites, restaurants	2008	$1.530	$1.000	2007		
	Comprehensive	2010					
Tennessee	Work sites	2007	$0.200	$0.070	2002	377	0
			$0.620	$0.420	2007		
Texas	Partial/none	—b	$1.410	$1.000	2007	244	0
Utah	Work sites, restaurants	2006	$0.695	$0.180	2002	35	145
	Comprehensive	2009	$1.70	$1.005	2010		
Vermont	Comprehensive	2009	$0.930	$0.490	2002	409	23
			$1.190	$0.260	2003		
			$1.790	$0.600	2006		
			$1.990	$0.200	2008		
			$2.240	$0.250	2009		
			$2.620	$0.380	2011		
			$2.750	$0.130	2014		
Virginia	Partial/none	—b	$0.200	$0.175	2004	1100	0
			$0.300	$0.100	2005		
Washington	Comprehensive	2005	$1.425	$0.600	2002	145	49
			$2.025	$0.600	2005		
			$3.025	$1.000	2010		
West Virginia	Partial/none	—b	$0.550	$0.380	2003	224	0
Wisconsin	Comprehensive	2010	$0.770	$0.180	2001	327	0
			$1.770	$1.000	2008		
			$2.520	$0.750	2009		
Wyoming	Partial/none	—b	$0.600	$0.480	2003	400	0

a “Comprehensive” indicates a law that prohibits smoking in all indoor areas of private work sites, restaurants, and bars. Smoke-free restrictions in 1 or 2 of these 3 areas — private work sites, restaurants, bars — are indicated by listing the places where smoking is prohibited. “Partial/none” indicates a law that does not prohibit smoking in all indoor areas but permits it in designated smoking areas or separately ventilated areas or no smoke-free law.

b Year of relevant smoke-free law or excise tax increase or decrease is not applicable because the state did not implement a smoke-free law or excise tax increase or decrease from 2000 through 2014.

Year	Total Statewide Comprehensive Smoke-Free Laws in Effect
2000	0
2001	0
2002	1
2003	2
2004	3
2005	5
2006	9
2007	13
2008	16
2009	22
2010	26
2011	26
2012	27
2013	27
2014	27

By December 31, 2009, 10 states had laws in effect that prohibited smoking in all indoor areas of work sites, restaurants, or bars but did not prohibit all smoking in all 3 areas. From 2000 through 2009, 19 states made no changes to their smoking laws.

In 2010, comprehensive smoke-free laws went into effect in 4 additional states (Kansas, Michigan, South Dakota, and Wisconsin). No new comprehensive state smoke-free laws went into effect in 2011, and in 2012 a comprehensive smoke-free law went into effect in North Dakota. From 2012 through 2014, no state passed a comprehensive smoke-free law (Figure 1).

As of December 31, 2014, 24 states had no comprehensive smoke-free laws in place; 11 states had not changed their smoking laws since before 2000 and had no laws partially or totally restricting smoking.

As of December 31, 2014, statewide comprehensive smoke-free laws, by US Census region, ranged from 18% of the 17 states in the South region to 83% in the Midwest region (Table 2). From 2000 through 2009, 6 southern states (Arkansas, Delaware, Florida, Louisiana, Maryland, and Tennessee) and the District of Columbia had laws go into effect that created more stringent smoking restrictions in public places (Table 1). The most stringent laws were comprehensive smoke-free laws that went into effect in Delaware (2002), the District of Columbia (2007) and Maryland (2008). Less stringent restrictions also went into effect, prohibiting smoking in at least one location (work sites, restaurants, or bars) in 4 southern states (Arkansas, Florida, Louisiana, and Tennessee). Between 2010 and 2014, North Carolina was the only southern state to change its statewide smoking prohibitions, prohibiting smoking in 2 locations (restaurants and bars) in 2010. No southern state has had a comprehensive statewide smoke-free law go into effect since 2008.

**Table 2:** State Smoke-Free Laws and State Cigarette Excise Tax Rates Per Pack, by State and US Census Region, 2014

US Census Region	Type of Smoke-Free Law^a^	State Cigarette Excise Tax Rate, Per Pack
**Northeast **		
Connecticut	Partial/none	$3.400
Maine	Comprehensive	$2.000
Massachusetts	Comprehensive	$3.510
New Hampshire	Restaurants	$1.780
New Jersey	Comprehensive	$2.700
New York	Comprehensive	$4.350
Pennsylvania	Work sites	$1.600
Rhode Island	Comprehensive	$3.500
Vermont	Comprehensive	$2.750
Average for region	$2.84	
**South **		
Alabama	Partial/none	$0.425
Arkansas	Work sites	$1.150
Delaware	Comprehensive	$1.600
District of Columbia	Comprehensive	$2.900
Florida	Work sites, restaurants	$1.339
Georgia	Partial/none	$0.370
Kentucky	Partial/none	$0.600
Louisiana	Work sites, restaurants	$0.360
Maryland	Comprehensive	$2.000
Mississippi	Partial/none	$0.680
North Carolina	Restaurants, bars	$0.450
Oklahoma	Partial/none	$1.030
South Carolina	Partial/none	$0.570
Tennessee	Work sites	$0.620
Texas	Partial/none	$1.410
Virginia	Partial/none	$0.300
West Virginia	Partial/none	$0.550
Average for region	$0.96	
**Midwest**		
Illinois	Comprehensive	$1.980
Indiana	Work sites, restaurants	$0.995
Iowa	Comprehensive	$1.360
Kansas	Comprehensive	$0.790
Michigan	Comprehensive	$2.000
Minnesota	Comprehensive	$2.830
Missouri	Partial/none	$0.170
Nebraska	Comprehensive	$0.640
North Dakota	Comprehensive	$0.440
Ohio	Comprehensive	$1.250
South Dakota	Comprehensive	$1.530
Wisconsin	Comprehensive	$2.520
Average for region	$1.38	
**West **		
Alaska	Partial/none	$2.000
Arizona	Comprehensive	$2.000
California	Partial/none	$0.870
Colorado	Comprehensive	$0.840
Hawaii	Comprehensive	$3.200
Idaho	Restaurants	$0.570
Montana	Comprehensive	$1.700
Nevada	Work sites, restaurants	$0.800
New Mexico	Comprehensive	$1.660
Oregon	Comprehensive	$1.310
Utah	Comprehensive	$1.700
Washington	Comprehensive	$3.025
Wyoming	Partial/none	$0.600
Average for region	$1.56	

a “Comprehensive” indicates a law that prohibits smoking in all indoor areas of private work sites, restaurants, and bars. Smoke-free restrictions in 1 or 2 of these 3 areas — private work sites, restaurants, bars — are indicated by listing the places where smoking is prohibited. “Partial/none” indicates a law that does not prohibit smoking in all indoor areas but permits it in designated smoking areas or in separately ventilated areas, or state has no smoke-free law.

**Figure 2:**
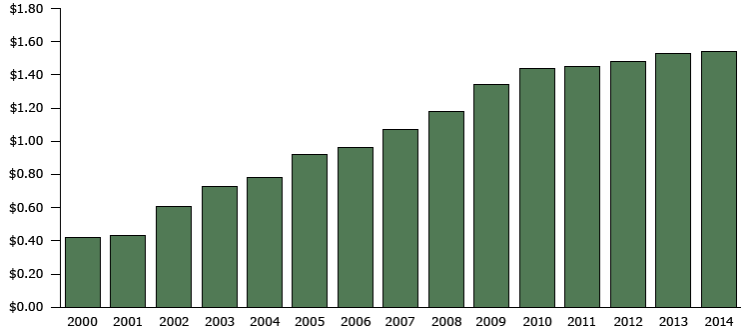
State cigarette excise tax laws and the national average state cigarette excise tax rate per pack in effect, by year, 2000–2014. Source: CDC’s State Tobacco Activities Tracking and Evaluation System. **Table:** 

From 2000 through 2009, the average state cigarette excise tax increased by $0.92 per pack, from $0.42 in 2000 to $1.34 in 2009 (Figure 2). During this period, 46 states and the District of Columbia increased their state excise tax rates at least once, and 102 laws went into effect that increased excise taxes (Table 1). Only 4 states — California, Missouri, North Dakota, and South Carolina — did not change their excise tax rate during this period. The largest number of state cigarette excise tax increases (in 21 states) occurred in 2002. These 21 laws produced the largest annual increase in the national average state excise tax rate — $0.18 (Figure 2).

Year	National Average State Cigarette Excise Tax Per Pack
2000	$0.42
2001	$0.43
2002	$0.61
2003	$0.73
2004	$0.78
2005	$0.92
2006	$0.96
2007	$1.07
2008	$1.18
2009	$1.34
2010	$1.44
2011	$1.45
2012	$1.48
2013	$1.53
2014	$1.54

From 2000 through 2009, 23 states and the District of Columbia increased their cigarette excise tax rates by $1.00 or more (Table 1). By December 31, 2009, 19 states and the District of Columbia had cigarette excise tax rates of $1.50 or more (Table 1). Furthermore, 2 states increased their cigarette excise taxes by over $2.00 during the same period: Connecticut implemented 4 increases totaling $2.50, and Rhode Island implemented 5 increases totaling $2.75.

From January 1, 2010, through December 31, 2014, the national average cigarette excise tax increased by $0.20 per pack, from $1.34 to $1.54 (Figure 2). During this period, 14 states and the District of Columbia increased their state excise tax rates at least once. Changes in the average excise tax rate during this period peaked in 2010, when 6 states raised their cigarette excise tax (Table 1). State legislative activity in this area slowed after 2010; for example, only 3 states implemented increases in 2014. One of these states (Oregon) increased its excise tax by $0.13, reversing a 2004 $0.10 decrease in the excise tax per pack, thus effectively raising the excise tax by only $0.03 per pack.

Between 2010 and 2014, only 6 states increased their excise tax rate by $1.00 or more; 2 of those states, Minnesota and New York, increased their excise tax rates by $1.60. Eleven states did not increase their cigarette excise tax rates from 2004 to 2014. Tax rates in 4 of these states — California, Missouri, Nevada, and North Dakota — were unchanged since before 2000. Furthermore, no state in the southern region increased its cigarette excise tax rate from 2010 (when South Carolina increased its excise tax by $0.50, to $0.57 per pack) through 2014 (Table 1). In contrast, 6 northeastern states increased their cigarette excise tax from 2010 through 2014 (Connecticut by $0.40 in 2011, Massachusetts by $1.00 in 2013, New Hampshire by $0.10 in 2013 (to reverse a $0.10 decrease in 2011), New York by $1.60 in 2010, Rhode Island by $0.04 in 2012, and Vermont by $0.38 in 2011 and $0.13 in 2014) (Table 1).

## Discussion

The findings from this study indicate that 2000 to 2009 was a decade of major progress in state implementation of comprehensive smoke-free laws and cigarette excise tax increases. However, progress has slowed considerably since 2009. Only one state, (North Dakota) had a comprehensive smoke-free law go into effect between 2012 and 2016; on June 9, 2016, new California provisions go into effect that make the state’s smoke-free law comprehensive. Twenty-four states currently have minimal or no statewide smoking restrictions in effect. Increases in state cigarette excise taxes also slowed; between 2010 and 2014, 14 states and the District of Columbia had excise tax increases take effect. In contrast, 14 states and the District of Columbia had increases take effect in 2009 alone. If progress in implementing these evidence-based interventions does not resume, the United States may not achieve its *Healthy People 2020* objectives to establish smoke-free indoor air laws in all 50 states and the District of Columbia, and to increase cigarette excise taxes by at least $1.50 per pack in all 50 states and the District of Columbia by the end of the current decade (objectives TU13 and TU-17.1, respectively) (10). This stalled progress in both policy areas may contribute to excess, preventable tobacco-related illness and death (1).

Comprehensive smoke-free laws reduce exposure to secondhand smoke, help smokers quit, and improve health outcomes; these laws typically have high levels of public support and compliance and do not have an adverse economic impact on the hospitality industry (eg, restaurants, bars) (1,2,8,11). However, this strong evidence base has not translated into continued progress in state implementation of comprehensive smoke-free laws in recent years, although such progress has continued at the local level. With a few exceptions, state laws adopted in more recent years are more often comprehensive laws, rather than partial laws (12). Research indicates that once states pass partial smoking restrictions, they are slow to revisit these laws and strengthen them (1,12).

The Institute of Medicine, the US Surgeon General, and the World Health Organization all agree that increasing the price of tobacco products is the single most effective way to reduce tobacco use (1,13,14). Increases in the price of tobacco products, including increases through state excise taxes, prevent smoking initiation, promote smoking cessation, and reduce the prevalence and intensity of tobacco use by adolescents and adults, thereby improving health outcomes (1,15–17). The recent slowed progress in cigarette excise tax increases is significant for 3 reasons. First, state excise tax rates vary widely — from $0.17 per pack in Missouri to $4.35 per pack in New York (1). Without sustained progress in increasing state excise taxes, especially in states where such taxes are low, low cigarette prices will contribute to smoking-related health disparities between states. State variations in cigarette tax rates and prices encourage tax avoidance and evasion, decreasing state revenues and undercutting health protection by perpetuating access to less expensive cigarettes (1). Second, excise taxes are levied as static dollar amounts per unit; if not periodically increased, these amounts do not keep pace with inflation (1). Third, the tobacco industry often responds to tax increases with promotions, such as offering coupons and discounts, to offset the projected decline in cigarette use (1,18). These 3 factors undermine the impact of tax increases on public health, especially when those increases are infrequent or small.

This study also highlights clear regional disparities. With the exception of Delaware, the District of Columbia, and Maryland, the remaining 14 states in the southern US Census region lack comprehensive smoke-free protections at the state level (Table 2). As of December 31, 2014, nine of these states either did not have any statewide smoking restrictions or had laws requiring only designated smoking areas or separately ventilated areas in work sites, restaurants, and bars (Table 2). Except for Delaware, the District of Columbia, and Maryland, North Carolina is the only southern state with a state law prohibiting smoking in restaurants and bars (Table 2). Furthermore, the average state excise tax for southern states is $0.96 per pack, which is considerably lower than the national average of $1.54 per pack (5). In addition to often lacking comprehensive smoke-free laws and high excise taxes, southern states also tend to provide low levels of funding for state tobacco control programs and to experience smoking prevalence and smoking-related diseases at higher rates than other regions (1,19–22). In the absence of more consistent state implementation of evidence-based tobacco control interventions across regions, these regional health disparities are likely to persist (1).

Despite stalled progress at the state level, progress continued in implementing comprehensive smoke-free laws at the local level. At least 697 localities have ordinances in place that prohibit smoking in restaurants, work sites, and bars, and this number continued to increase during the same period when state progress in this area stalled (23,24). Local progress in this area occurred in numerous states that lack comprehensive state laws, including in several southern states. In some southern states, such as Kentucky, Mississippi, South Carolina, Texas, and West Virginia, a substantial portion of the state’s population lives under comprehensive local smoke-free laws (25,26). Experience suggests that a critical mass of comprehensive local smoke-free laws in a state may lay the groundwork for the eventual adoption of a statewide comprehensive law (8,12). However, in 8 states that lack comprehensive state smoke-free laws, local jurisdictions are preempted from enacting smoking restrictions that are more restrictive than state law in some or all settings (7). Examining how local smoking restrictions evolved in recent years and their impact on public health merits further research. However, statewide legislation remains the only way to ensure that local laws do not lead to disparities in protections between localities.

The patterns observed in the local and state policy domains could be the result of multiple factors. For example, tobacco control efforts in some states may give priority to local policies over state policies in one or both of these domains because of the greater challenges associated with making progress at the state level and in the hope that local progress could eventually lay the groundwork for state progress. Another contributing factor could be the emergence of electronic cigarettes and other electronic nicotine delivery systems (27). The rapid spread and increasing use of these novel products may contribute to the stall in adopting additional state smoke-free laws, because decision makers may be uncertain whether to include these products in smoking restrictions, or they may perceive them as complicating implementation and enforcement of smoke-free laws (27). Another potential explanation is the recent reduction in funding for state tobacco prevention and control programs (20,28). The stall in adopting smoke-free laws may also be due, in part, to longstanding and continued efforts by the tobacco industry to interfere with the implementation of these efforts at the state and local levels (29).

The findings in this study are subject to at least 5 limitations. First, the study considered only comprehensive smoke-free policies and not less restrictive policies that provide some level of protection from secondhand smoke. For example, California has substantial statewide smoking restrictions that were enacted in 1994, at an early point in the evolution of smoke-free policies, but they do not meet current definitions of comprehensive smoke-free laws. The state has recently updated these provisions to enact a law making California comprehensively smoke-free, effective June 9, 2016. Second, although progress in enacting smoke-free legislation at the local level continued, local smoke-free ordinances were not taken into account in this study. Third, our analysis does not include information on the price per pack of cigarettes, which varies considerably from state to state, even between states with similar excise tax rates, because of differences in manufacturer, wholesaler, and retailer pricing and discounting practices. Fourth, state excise taxes were examined for cigarettes only, excluding taxes on other tobacco products. In general, state cigarette excise taxes tend to be substantially higher than state taxes on other tobacco products (7). Increasing the price of cigarettes without increasing the price of other combustible tobacco products may lead smokers to switch from an expensive tobacco product to a cheaper one instead of quitting all tobacco use and may lead adolescents to initiate tobacco use with cheaper products, such as little cigars (30). Finally, the study did not capture administrative decisions, including rule-making, opinions of attorneys general, or court decisions.

Recent slowdowns in implementing new statewide comprehensive smoke-free laws and state excise taxes may jeopardize progress in reducing tobacco use and the health and economic burdens it imposes. Although some progress was made at the local level, the first half of the current decade (2010–2014) was marked by a lack of momentum in both of these key policy areas at the state level, especially when compared with the considerable progress from 2000 through 2009. This stall also highlights regional disparities in tobacco prevention and control, particularly the lack of progress in the South. Without accelerated progress in implementing both of these important interventions, efforts to reduce tobacco use, exposure to secondhand smoke, health disparities, and tobacco-related illness and death in the United States could be undermined.
